# The Statesboro Lynching

**Published:** 1904-09

**Authors:** 


### THE STATESBORO LYNCHING.

THE Statesboro lynching presents psychological phases that are interesting to the sociologist and physician. There was the spectacle of two negroes tried and convicted of a hideous crime by due process of law made the object of the blood lust of the mob whose regard for the vaunted majesty of the law was equal to that of their victims. Vengeance is a primitive emotion, law a secondary convention, and when conventional habiliments are stripped off, the man is as raw and naked now as in the stone age. It is a curious commentary on the world's progress since its infancy that in a moment the rouged check of effete civilization changes to the painted face of the savage.

One can not but think of Truthful James—

"Do I sleep? do I dream?
Do I wander and doubt?
Is things what they seem,
Or is visions about?
Is our civilization a failure,
And is the Caucasian played out?"

If education and advancement in intellectual cultivation teach anything, it is self-restraint, the trained ability to control one's self to the inhibition of primitive emotions. Events like the Statesboro affair show how faint is the impress of modernity. Here in Georgia we do not care for the remarks of unfriendly critics. This is a matter of indifference. But we have a sense of mortification that such things are committed by our own people and in our very midst.

